# Distinct Expression Profiles of Neuroblastoma-Associated mRNAs in Peripheral Blood and Bone Marrow of Non-High-Risk and High-Risk Neuroblastoma Patients

**DOI:** 10.3390/biology13050345

**Published:** 2024-05-15

**Authors:** Naoko Nakatani, Kaung Htet Nay Win, Cho Yee Mon, Tomoko Fujikawa, Suguru Uemura, Atsuro Saito, Toshiaki Ishida, Takeshi Mori, Daiichiro Hasegawa, Yoshiyuki Kosaka, Shotaro Inoue, Akihiro Nishimura, Nanako Nino, Akihiro Tamura, Nobuyuki Yamamoto, Kandai Nozu, Noriyuki Nishimura

**Affiliations:** 1 Department of Pediatrics, Kobe University Graduate School of Medicine, Kobe 650-0017, Japan; nknaka@med.kobe-u.ac.jp (N.N.); tomoko14@med.kobe-u.ac.jp (T.F.); stinoue@med.kobe-u.ac.jp (S.I.); ak2213@med.kobe-u.ac.jp (A.N.); atamura@med.kobe-u.ac.jp (A.T.); nyama@med.kobe-u.ac.jp (N.Y.); nozu@med.kobe-u.ac.jp (K.N.); 2 Department of Public Health, Kobe University Graduate School of Health Science, Kobe 654-0142, Japan; kaunghtetnaywin.tetsuya@gmail.com (K.H.N.W.); choyeemon91@gmail.com (C.Y.M.); 3 Department of Hematology/Oncology, Kobe Children’s Hospital, Kobe 650-0047, Japan; sguemura_kch@hp.pref.hyogo.jp (S.U.); atsurocytosis@mac.com (A.S.); ishida_kch@hp.pref.hyogo.jp (T.I.); tsmori_kch@hp.pref.hyogo.jp (T.M.); hasegawa_kch@hp.pref.hyogo.jp (D.H.); kosaka_kch@hp.pref.hyogo.jp (Y.K.); n.w.n.nino@gmail.com (N.N.)

**Keywords:** high-risk (HR) neuroblastoma (NB), non-high-risk (non-HR) neuroblastoma (NB), neuroblastoma-associated mRNAs (NB-mRNAs), peripheral blood (PB), bone marrow (BM)

## Abstract

**Simple Summary:**

Neuroblastoma (NB) is a common pediatric solid tumor characterized by extreme heterogeneity; non-high-risk (non-HR) patients have near uniform survival and HR patients experience a fatal demise. Metastatic disease is the principal cause of death among both non-HR and HR NB patients. Previous studies have reported a significant but limited prognostic value of quantitative PCR (qPCR)-based assays, measuring overlapping but different sets of neuroblastoma-associated mRNAs (NB-mRNAs), to detect metastatic disease in both non-HR and HR patient samples. A droplet digital PCR (ddPCR)-based assay measuring seven NB-mRNAs was developed and exhibited a better prognostic value for HR patient samples than qPCR-based assays. However, it remained to be tested on non-HR patient samples. In the present study, we employed the ddPCR-based assay to study peripheral blood (PB) and bone marrow (BM) samples collected at diagnosis from eight non-HR and eleven HR cases and characterized the expression profiles of NB-mRNAs. The most highly expressed NB-mRNAs in PB and BM differed between non-HR and HR cases. The expression levels of NB-mRNAs in PB and BM were 5 to 1000 times lower in non-HR cases than in HR cases. The PB to BM ratio of NB-mRNAs was 10 to 100 times higher in non-HR cases than in HR cases. The present case series suggests that non-HR and HR NB patients have the distinct expression profiles of NB-mRNAs in their PB and BM.

**Abstract:**

Non-high-risk (non-HR) neuroblastoma (NB) patients have excellent outcomes, with more than a 90% survival rate, whereas HR NB patients expect less than a 50% survival rate. Metastatic disease is the principal cause of death among both non-HR and HR NB patients. Previous studies have reported the significant but limited prognostic value of quantitative PCR (qPCR)-based assays, measuring overlapping but different sets of neuroblastoma-associated mRNAs (NB-mRNAs), to detect metastatic disease in both non-HR and HR patient samples. A droplet digital PCR (ddPCR)-based assay measuring seven NB-mRNAs (CRMP1, DBH, DDC, GAP43, ISL1, PHOX2B, and TH mRNAs) was recently developed and exhibited a better prognostic value for HR patient samples than qPCR-based assays. However, it remained to be tested on non-HR patient samples. In the present study, we employed the ddPCR-based assay to study peripheral blood (PB) and bone marrow (BM) samples collected at diagnosis from eight non-HR and eleven HR cases and characterized the expression profiles of NB-mRNAs. The most highly expressed NB-mRNAs in PB and BM differed between non-HR and HR cases, with the CRMP1 mRNA being predominant in non-HR cases and the GAP43 mRNA in HR cases. The levels of NB-mRNAs in PB and BM were 5 to 1000 times lower in non-HR cases than in HR cases. The PB to BM ratio of NB-mRNAs was 10 to 100 times higher in non-HR cases compared to HR cases. The present case series suggests that non-HR and HR NB patients have the distinct expression profiles of NB-mRNAs in their PB and BM.

## 1. Introduction

Neuroblastoma (NB) is a pediatric solid tumor that accounts for approximately 10% of all pediatric cancers and up to 15% of pediatric cancer deaths [1,2]. NB originates from the neural crest during the embryonic stage and exhibits extreme heterogeneity, ranging from spontaneous regression to highly malignant progression [3,4]. NB patients have very different prognoses, one with near uniform survival and the other with fatal demise. Consequently, they have been stratified for decades into low-risk (LR), intermediate-risk (IR), and high-risk (HR) groups by several risk classifiers worldwide [5,6,7,8,9,10,11,12].

LR and IR (non-HR) patients generally have excellent outcomes, with an overall survival rate exceeding 90%, leading to a reduction in the current treatment protocols [8,13]. In contrast, HR patients are far from achieving optimal outcomes. Although HR patients received multimodal standard therapy, including multi-agent induction chemotherapy, surgery, radiotherapy, and high-dose chemotherapy with autologous peripheral blood stem cell rescue, less than 50% of them only achieve long-term survival [14,15,16]. Up to 20% of HR patients have residual disease that results in refractory or progressive disease, while the remainder may achieve remission; however, more than 50% have residual disease that ultimately leads to relapse [17].

Metastatic disease is the principal cause of death among both non-HR and HR NB patients. Within the International Neuroblastoma Risk Group (INRG) classification system [6], it is evaluated in the INRG staging system [7], along with other statistically significant prognostic factors, including age, histologic category, grade of tumor differentiation, ploidy, MYCN amplification, and 11q aberration. Approximately 50% of newly diagnosed NB patients with metastatic disease (stage M), except for less than 18 months of age without MYCN amplification, are stratified into the HR group. The remaining 50% with localized disease (stage L), excluding those with MYCN amplification, are categorized as the non-HR group. However, some non-HR patients experience relapse and succumb to the disease [13].

In order to achieve the precise detection of metastatic disease, overlapping but different sets of neuroblastoma-associated mRNAs (NB-mRNAs) that are selected by their ability to define a cut-off value between control and NB patient samples have been measured by quantitative PCR (qPCR) for decades, due to the absence of a common genetic aberration in NB cells [18,19]. However, previous studies have reported the significant, but limited, prognostic value of qPCR-based assays to detect NB cells in peripheral blood (PB) and bone marrow (BM) of both non-HR and HR patients [20,21,22,23,24,25,26,27]. For non-HR patient samples, the PHOX2B mRNA [27], DCX and TH mRNAs [20], DCX, PHOX2B, and TH mRNAs [26], and CHRNA3, DDC, GAP43, PHOX2B, and TH mRNAs [24] were used as NB-mRNAs. For HR patient samples, the following NB-mRNAs were reported: DCX, PHOX2B, and TH mRNAs [22], B4GALNT1, CCND1, ISL1, and PHOX2B mRNAs [23], CHRNA3, DDC, GAP43, PHOX2B, and TH mRNAs [21], and CHGA, DCX, DDC, PHOX2B, and TH mRNAs [25]. Among the ten different NB-mRNAs, six NB-mRNAs (CHRNA3, DCX, DDC, GAP43, PHOX2B, and TH mRNAs) were common to both non-HR and HR patient samples, while four NB-mRNAs (B4GALNT1, CCND1, CHGA, and ISL1 mRNAs) were specific to HR patient samples.

We have recently developed a droplet digital PCR (ddPCR)-based assay measuring seven NB-mRNAs (CRMP1, DBH, DDC, GAP43, ISL1, PHOX2B, and TH mRNAs; 7NB-mRNAs ddPCR assay), to detect NB cells in HR patient samples [28]. CRMP1 and GAP43 genes were implicated in growth cone remodeling during neuronal development and regeneration; DBH, DDC, and TH genes were found to encode enzymes participating in the synthesis of catecholamines; and ISL1 and PHOX2B genes were found to encode transcription factors involved in the development of motor and noradrenergic neurons. In terms of the recent NB classification, in which NB cells were classified into either the ADRN type, associated with a sympathetic neuronal cell state, or the MES type, associated with an undifferentiated neural crest-like cell state, CRMP1, DBH, DDC, GAP43, ISL1, PHOX2B, and TH genes were all classified into ADRN-type genes [29,30,31]. Although the 7NB-mRNAs ddPCR assay exhibited an improved prognostic value for HR patient samples [28,32], it remained to be tested on non-HR patient samples.

Although non-HR patients can expect near uniform survival, some of them will suffer from a relapse and succumb to the disease [13]. Given that metastatic disease is the principal cause of death and the most fundamental factor in the INRG risk classification system [6], it may be due to the inability to detect metastatic disease in non-HR patients. We hypothesized that some “HR” patients with metastatic diseases may be mistakenly stratified into a “non-HR” group due to the inability of qPCR-based assays to detect metastatic disease. To avoid this risk stratification error, the 7NB-mRNAs ddPCR assay may provide a promising option to detect metastatic diseases in non-HR patients. In the present study, we determined the levels of seven NB-mRNAs in PB and BM at diagnosis for eight non-HR and eleven HR cases by ddPCR and characterized the expression profiles of NB-mRNAs.

## 2. Materials and Methods

### 2.1. Patients and Samples

Patients were diagnosed and stratified into very-low-risk (VLR), LR, IR, or HR groups, according to the INRG classification system [6,7]. They were treated at Kobe Children’s Hospital or Kobe University Hospital between June 2012 and October 2018. PB, BM, and urine samples were collected at diagnosis, with written informed consent. Vanillylmandelic acid (VMA) and homovanillic acid (HVA) levels in urine and neuron-specific enolase (NSE), lactate dehydrogenase (LDH), and ferritin levels in serum were extracted from the patient’s medical records. Normal reference ranges for VMA, HVA, NSE, LDH, and ferritin were 1.2–4.9 μg/mg creatinine, 1.6–5.5 μg/mg creatinine, 0–16.3 ng/mL, 124–270 IU/L, and 6–138 ng/mL, respectively, for most children. This study received ethical approval from the ethics committee at Kobe Children’s Hospital (No. 31-91) and Kobe University Graduate School of Medicine (No. 180278), and it adhered to the guidelines for Clinical Research by Kobe University Graduate School of Medicine.

### 2.2. RNA Isolation and cDNA Synthesis

PB and BM sample preparation followed the method previously described [28]. The PB and BM samples were separated using the Mono-Poly resolving medium (DS Pharma Biomedical, Osaka, Japan), and nucleated cells were collected. Total RNA was extracted with a TRIzol Plus RNA purification kit (Life Technologies, Carlsbad, CA, USA). The cDNA was synthesized from 1 or 0.5 μg of total RNA using a Quantitect Reverse Transcription Kit (Qiagen, Valencia, CA, USA), and stored at −80 °C until use.

### 2.3. 7NB-mRNAs ddPCR Assay

A ddPCR-based assay measuring 7 NB-mRNAs (7NB-mRNAs ddPCR assay) was developed to achieve more precise detection of NB cells than qPCR-based assays [28]. The 7 NB-mRNAs were selected based on their specific expression in sphere NB cells enriched in chemoresistant cancer stem-like NB cells compared to parental NB cells [28,33]. Briefly, the expression levels of 7 NB-mRNAs (CRMP1, DBH, DDC, GAP43, ISL1, PHOX2B, and TH mRNAs) and a reference gene mRNA (HPRT1 mRNA) were quantitated using the QX200 ddPCR system (Bio-Rad Laboratories, Hercules, CA, USA), according to the digital MIQE (Minimum Information for Publication of Quantitative Digital PCR Experiments) guidelines [34,35]. The level of 7NB-mRNAs (combined signature) was defined as the weighted sum of 7 relative copy numbers (level of each NB-mRNA), in which the reciprocal of the 90th percentile in the non-NB PB and BM control samples was used for the weighting of each NB-mRNA [28].

## 3. Results

### 3.1. Characteristics of Non-HR and HR Cases

In the present study, all newly diagnosed NB cases, between June 2012 and October 2018, at Kobe Children’s Hospital and Kobe University Hospital were included. Cases without available PB and BM samples for the 7NB-mRNAs ddPCR assay were excluded. Eight non-HR (comprising two VLR, two LR, and four IR) cases were compared with eleven HR cases, as shown in Table 1. These non-HR and HR cases displayed typical non-HR and HR characteristics of the respective groups. The ages of the patients ranged from 4 to 68 months (median 12) for non-HR cases and 11 to 56 months (median 30) for HR cases. BM metastasis occurred in 13% (1/8) of non-HR cases and 91% (10/11) of HR cases. MYCN amplification was absent in non-HR cases (0/8) and present in 55% (6/11) of HR cases. Following a minimum of 48 months of follow-up, all non-HR cases (8/8) were alive without relapse, whereas 73% (8/11) of HR cases had relapsed and died.

### 3.2. Levels of Seven NB-mRNAs in PB and BM Samples of Non-HR and HR Cases

**Figure 1:**
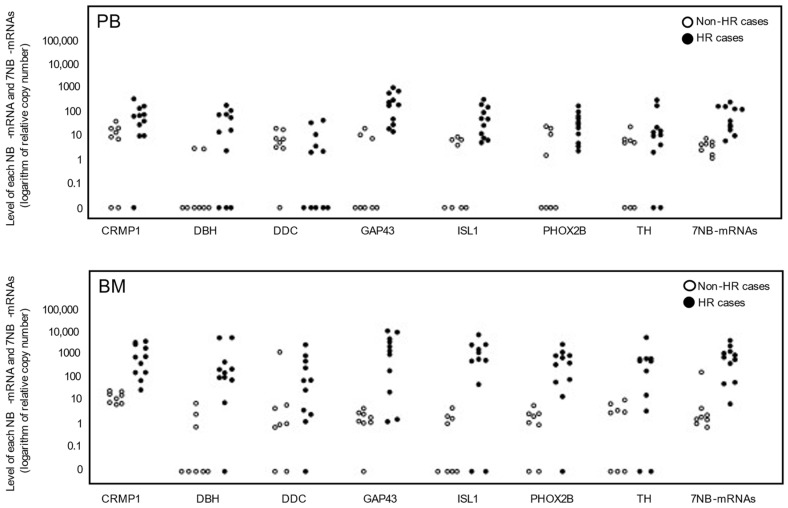
Levels of 7 NB-mRNAs (CRMP1, DBH, DDC, GAP43, ISL1, PHOX2B, and TH mRNAs) in PB and BM samples of non-HR and HR cases. A total of 19 PB and 19 BM samples for 8 non-HR and 11 HR cases were subjected to 7NB-mRNAs ddPCR assay. NB-mRNA, neuroblastoma-associated mRNA; PB, peripheral blood; BM, bone marrow; HR, high risk; open circle, non-HR case; closed circle, HR case; CRMP1, collapsin response mediator protein 1; DBH, dopamine beta-hydroxylase; DDC, dopa decarboxylase; GAP43, growth-associated protein 43; ISL1, ISL LIM homeobox 1; PHOX2B, paired-like homeobox 2b; TH, tyrosine hydroxylase.

The levels of seven NB-mRNAs (CRMP1, DBH, DDC, GAP43, ISL1, PHOX2B, and TH mRNAs) in the PB and BM samples from non-HR and HR cases were determined by ddPCR and are plotted in Figure 1. The study evaluated 19 PB and 19 BM samples from 8 non-HR and 11 HR cases. Although each NB-mRNA was not detected in 3–9 PB and 0–6 BM samples, 7NB-mRNAs were detected in all PB and BM samples (Figure 1). The most highly expressed NB-mRNAs in PB and BM differed between non-HR and HR cases: the CRMP1 mRNA was the most expressed in PB and BM of non-HR cases, while the GAP43 mRNA was the most expressed in PB and BM of HR cases (Table 2). Consistent with the pathological detection of BM metastasis (Table 1), the levels of seven NB-mRNAs in BM were approximately 10 to 1000 times lower in non-HR cases than HR cases (Table 2). Similarly, the levels of seven NB-mRNAs in PB were also about 5 to 50 times lower in non-HR cases compared to HR cases, with the exception of the DDC mRNA (Table 2). In contrast, the PB to BM ratio (PB/BM ratio) of seven NB-mRNAs was approximately 10 to 100 times higher in non-HR cases than in HR cases, except for the GAP43 mRNA (Table 2).

**Figure 2:**
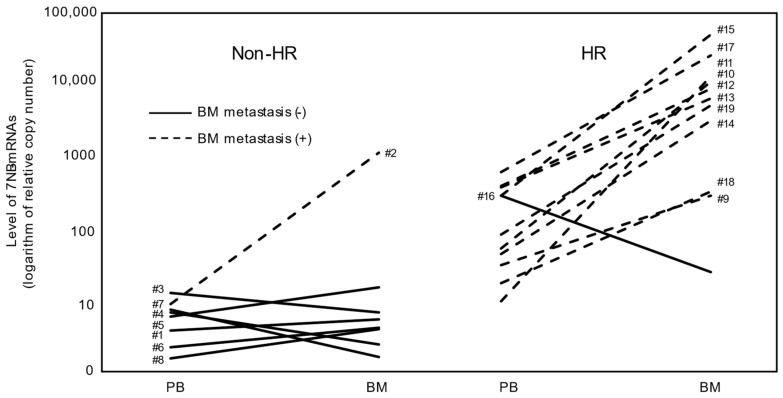
Levels of 7NB-mRNAs in PB and BM samples for each non-HR and HR case. Each non-HR (#1–#8) and HR (#9–#19) case was plotted as a solid or dashed line. Characteristics of #1–#19 are described in Table 1. NB-mRNA, neuroblastoma-associated mRNA; PB, peripheral blood; BM, bone marrow; HR, high risk; solid lines, with BM metastasis at diagnosis; dashed lines, without BM metastasis at diagnosis.

To examine the PB/BM ratio for each non-HR and HR case, the levels of 7NB-mRNAs in PB and BM of each non-HR and HR case are plotted in Figure 2. The PB/BM ratio was consistently less than 1 in cases with BM metastasis (cases #2, #9–#15, and #17–#19). In cases without BM metastasis, the ratio varied, being less than 1 in some non-HR cases (cases #1, #5, #6, and #8) and greater than 1 in other non-HR cases (cases #3, #4, and #7), as well as one HR case (#16).

### 3.3. Levels of Tumor Markers in Non-HR and HR Cases

**Figure 3:**
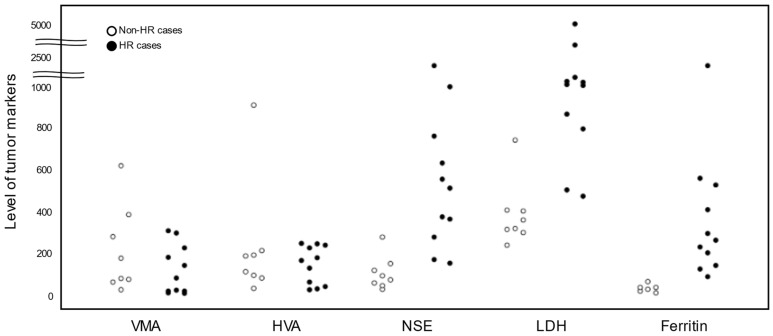
Levels of tumor markers (VMA, HVA, NSE, LDH, and ferritin) in non-HR and HR cases. NB-mRNA, neuroblastoma-associated mRNA; HR, high risk; PB, peripheral blood; BM, bone marrow; IQR, interquartile range; VMA, vanillylmandelic acid; HVA, homovanillic acid; NSE, neuron-specific enolase; LDH, lactate dehydrogenase.

While the INRG does not incorporate tumor markers (VMA, HVA, NSE, LDH, and Ferritin) into the risk factors [6,36,37], they are widely used as standard clinical evaluations for both non-HR and HR cases [38]. In the present study, the levels of tumor markers (VMA, HVA, NSE, LDH, and ferritin) in eight non-HR and eleven HR cases are plotted in Figure 3. Similar to the levels of the seven NB-mRNAs (Table 2), the levels of NSE, LDH, and ferritin were 3–8 times lower in non-HR cases than HR cases (Table 3). In contrast, VMA and HVA exhibited higher or similar levels in non-HR cases compared with HR cases (Table 3).

### 3.4. Association between Tumor Markers and 7NB-mRNAs

The association between the tumor markers and 7NB-mRNAs are shown in Table 4. Consistent with the previous observation in regard to the HR cases [39], correlations between the tumor markers and 7NB-mRNAs (i.e., >50% high seven NB-mRNAs in cases with high tumor markers and/or <50% high seven NB-mRNAs in cases with low tumor markers) were detected in both non-HR and HR cases. In non-HR cases, 7NB-mRNAs correlated with LDH in PB and VMA/HVA/NSE/LDH in BM. In HR cases, correlations were observed with VMA/NSE/LDH in PB and VMA/HVA/NSE/LDH in BM.

## 4. Discussion

In the present study, we determined the levels of seven NB-mRNA (CRMP1, DBH, DDC, GAP43, ISL1, PHOX2B, and TH mRNA) in PB and BM at diagnosis for eight non-HR and eleven HR cases by ddPCR and characterized their expression profiles. First, the most highly expressed NB-mRNAs in PB and BM differed between non-HR cases and HR cases (CRMP1 mRNA in PB and BM of non-HR cases and GAP43 mRNA in PB and BM of HR cases). Second, the expression levels of NB-mRNAs in PB and BM were 5 to 1000 times lower in non-HR cases than in HR cases. Third, the PB/BM ratio of NB-mRNAs was 10 to 100 times higher in non-HR cases than in HR cases.

The first finding that the most highly expressed NB-mRNAs in PB and BM of non-HR and HR cases were different (CRMP1 mRNA in PB and BM of non-HR cases and GAP43 mRNA in PB and BM of HR cases) might suggest different cell states of NB cells derived from non-HR and HR patients (Table 2). NB-mRNAs, which are predominantly expressed in NB cells, were selected based on their ability to define a cut-off value that distinguishes NB cells from normal cells [40]. In the present study, seven NB-mRNAs (CRMP1, DBH, DDC, GAP43, ISL1, PHOX2B, and TH mRNA) were used to detect NB cells. The differential but correlated expression of NB-mRNAs in PB and BM of HR patients has been recognized for decades, but definitive sets of NB-mRNAs for PB and BM samples have yet to be determined [18,25,28,41,42]. Additionally, novel mesenchymal (MES) NB-mRNAs (POSTN and PRRX1 mRNAs), as opposed to classical adrenergic (ADRN) NB-mRNAs (CHRNA3, DBH, DDC, PHOX2B, and TH mRNAs), have been reported to possess a prognostic power for peripheral blood stem cell (PBSC) samples [43]. Similarly, the optimal sets of NB-mRNAs for non-HR and HR patient samples might differ.

The second finding that the expression levels of NB-mRNAs in PB and BM were 5 to 1000 times lower in non-HR cases compared to HR cases, might reflect the metastatic tumor burden in NB patients (Table 2). To our knowledge, this is the first demonstration of the PB expression of NB-mRNAs being approximately 5 to 50 times lower in non-HR cases than in HR cases, indicative of fewer NB cells in PB. The finding that BM expression of NB-mRNAs is approximately 10 to 1000 times lower in non-HR cases compared to HR cases is consistent with the pathological detection of BM metastasis, 13% positive in non-HR cases versus 91% positive in HR cases (Table 1).

The third finding that the PB/BM ratio of NB-mRNAs was 10 to 100 times higher in non-HR cases than in HR cases, suggests that NB cells in non-HR patients may have a lower affinity to BM than those in HR cases (Table 2). Although the majority of PB and BM samples from non-HR patients did not show detectable NB-mRNAs by qPCR, all PB and BM samples from non-HR and HR cases exhibited detectable levels of the 7NB-mRNAs in the present study (Figure 1). This sensitive detection of NB-mRNAs by ddPCR, for the first time, enabled us to compare the PB/BM ratio of NB-mRNAs between non-HR and HR cases. The results revealed a difference of 10 to 100 times, indicating that NB cells in non-HR cases may have a lower affinity to BM compared to HR cases and may preferentially reside in PB.

However, the present study has certain limitations. The most critical issue is the small sample size that precludes conducting statistical analysis. It is also accompanied by the absence of events (relapse or death) in non-HR NB patients, hindering the prognostic evaluation of the 7NB-mRNAs ddPCR assay. An additional limitation is the heterogeneous sample size that means the obtained results are not sufficiently robust. Accordingly, the present findings should be interpreted with caution. We are now planning a larger-sized study, which will include enough events to validate the present findings.

## 5. Conclusions

The present case series suggests that non-HR and HR NB patients have the distinct expression profiles of NB-mRNAs in their PB and BM.

## Figures and Tables

**Table 1:** Characteristics of non-HR and HR cases.

Case	Risk	Age	Sex	Primary Tumor Site	Pathology	BM Metastasis	MYCN Status	DNA Ploidy	Relapse	Present Status
#1	IR	11 m	M	Adrenal grand	NB	(−)	non-Amp	Diploid and hyperdiploid	(−)	Alive
#2	LR	11 m	M	Adrenal grand	NB	(+)	non-Amp	Diploid and hyperdiploid	(−)	Alive
#3	LR	4 m	M	Adrenal grand	NB	(−)	non-Amp	Diploid and hyperdiploid	(−)	Alive
#4	VLR	68 m	M	Adrenal grand	GNB intermixed	(−)	non-Amp	Diploid	(−)	Alive
#5	IR	12 m	M	Adrenal grand	NB	(−)	non-Amp	Diploid	(−)	Alive
#6	IR	17 m	F	Mediastinum	NB	(−)	non-Amp	Diploid	(−)	Alive
#7	IR	9 m	M	Neck	NB	(−)	non-Amp	Diploid and hyperdiploid	(−)	Alive
#8	VLR	20 m	F	Adrenal grand	NB	(−)	non-Amp	Diploid and hyperdiploid	(−)	Alive
#9	HR	46 m	F	Adrenal grand	NB	(+)	Amp	Diploid	(+)	Dead
#10	HR	11 m	M	Adrenal grand	NB	(+)	Amp	Diploid	(+)	Dead
#11	HR	14 m	M	Adrenal grand	NB	(+)	Amp	Diploid	(+)	Dead
#12	HR	17 m	F	Adrenal grand	NB	(+)	Amp	Diploid	(+)	Dead
#13	HR	25 m	M	Adrenal grand	GNB nodular	(+)	non-Amp	Diploid	(+)	Dead
#14	HR	30 m	M	Adrenal grand	NB	(+)	non-Amp	Diploid	(+)	Dead
#15	HR	36 m	M	Adrenal grand	GNB nodular	(+)	non-Amp	Diploid	(−)	Alive
#16	HR	39 m	F	Neck	NB	(−)	non-Amp	Diploid	(+)	Dead
#17	HR	40 m	M	Adrenal grand	GNB nodular	(+)	Amp	Diploid	(−)	Alive
#18	HR	56 m	F	Mediastinum	NB	(+)	non-Amp	Diploid	(+)	Dead
#19	HR	19 m	M	Adrenal grand	NB	(+)	Amp	Diploid and near-tetraploid	(−)	Alive

HR, high risk; IR, intermediate risk; LR, low risk; VLR, very low risk; m, months; M, male; F, female; NB, neuroblastoma; GNB, ganglioneuroblastoma; BM, bone marrow; MYCN, MYCN proto-oncogene bHLH transcription factor; Amp, amplification; N/E, not examined.

**Table 2:** Levels of NB-mRNAs in non-HR and HR cases.

NB-mRNAs	Non-HR Cases			HR Cases		
	PB	BM	PB/BM Ratio	PB	BM	PB/BM Ratio
	Median	Median	Median	Median	Median	Median
	(IQR)	(IQR)	(IQR)	(IQR)	(IQR)	(IQR)
*CRMP1*	16.6	48.7	0.23	105.3	4349.4	0.02
	(7.7–30.7)	(24.4–71.0)	(0.08–0.60)	(29.2–180.0)	(759.7–15348.1)	(0.01–0.04)
*DBH*	0.0	0.0	0.61	25.3	734.2	0.01
	(0.0–1.0)	(0.0–2.8)	(0.31–1.53)	(1.60–123.9)	(372.4–1798.5)	(0.00–0.10)
*DDC*	8.7	2.1	1.45	2.7	307.7	0.01
	(4.4–15.0)	(1.1–14.4)	(0.14–2.64)	(0.0–10.8)	(8.50–1989.1)	(0.00–0.13)
*GAP43*	0.0	3.8	0.00	333.3	8892.1	0.06
	(0.0–12.2)	(2.6–6.8)	(0.00–1.20)	(61.1–812.3)	(506.2–29655.8)	(0.02–2.67)
*ISL1*	2.8	1.1	0.48	78.8	3436.8	0.03
	(0.0–9.6)	(0.0–4.3)	(0.00–1.32)	(14.6–209.3)	(1512.0–13919.8)	(0.00–0.05)
*PHOX2B*	1.0	3.9	0.39	46.1	2213.8	0.02
	(0.0–20.1)	(1.5–7.0)	(0.00–0.90)	(12.3–93.5)	(299.1–5100.5)	(0.01–0.07)
*TH*	7.2	8.1	0.47	15.8	2712.3	0.02
	(0.0–9.3)	(0.0–13.0)	(0.27–0.85)	(4.25–29.1)	(33.3–3508.3)	(0.00–0.08)
7NB-mRNAs	5.7	4.3	0.64	65.0	4194.7	0.03
	(3.1–6.8)	(3.2–7.7)	(0.42–2.03)	(31.1–247.8)	(1170.2–7560.0)	(0.01–0.06)

NB-mRNA, neuroblastoma-associated mRNA; HR, high risk; PB, peripheral blood; BM, bone marrow; IQR, interquartile range; CRMP1, collapsin response mediator protein 1; DBH, dopamine beta-hydroxylase; DDC, dopa decarboxylase; GAP43, growth-associated protein 43; ISL1, ISL LIM homeobox 1; PHOX2B, paired-like homeobox 2b; TH, tyrosine hydroxylase.

**Table 3:** Levels of tumor markers in non-HR and HR cases.

Tumor Markers	Non-HR Cases	HR Cases
	Median	Median
	(IQR)	(IQR)
Urinary VMA	128.1	81.4
(μg/mg/cr)	(73.3–306.7)	(19.1–203.4)
Urinary HVA	149.8	165.9
(μg/mg/cr)	(92.5–197.9)	(52.5–232.7)
Serum NSE	83.8	513.0
(ng/mL)	(54.6–126.0)	(321.0–698.0)
Serum LDH	339.0	1225.0
(U/L)	(310.3–404.0)	(831.5–1732.5)
Serum Ferritin	32.3	263.0
(ng/mL)	(22.2–37.0)	(172.3–467.5)

HR, high risk; IQR, interquartile range; VMA, vanillylmandelic acid; HVA, homovanillic acid; NSE, neuron-specific enolase; LDH, lactate dehydrogenase.

**Table 4:** Association between 7NB-mRNAs and tumor markers.

		Non-HR Cases		HR Cases	
		PB	BM	PB	BM
		High 7NB-mRNAs §	High 7NB-mRNAs #	High 7NB-mRNAs †	High 7NB-mRNAs ‡
Urinary VMA (μg/mg/cr)	≥100	1/4 (25%)	3/4 (75%)	3/5 (60%)	3/5 (60%)
	<100	3/4 (75%)	1/4 (25%)	3/6 (50%)	3/6 (50%)
Urinary HVA (μg/mg/cr)	≥100	2/5 (40%)	3/5 (60%)	3/7 (43%)	5/7 (71%)
	<100	2/3 (67%)	1/3 (33%)	3/4 (75%)	1/4 (25%)
Serum NSE (ng/mL)	≥100	1/3 (33%)	2/3 (67%)	6/11 (55%)	6/11 (55%)
	<100	3/5 (60%)	2/5 (40%)	0/0 (0%)	0/0 (0%)
Serum LDH (U/L)	≥500	1/1 (100%)	1/1 (100%)	6/10 (60%)	6/10 (60%)
	<500	3/7 (43%)	3/7 (43%)	0/1 (0%)	0/1 (0%)
Serum Ferritin (ng/mL)	≥300	0/0 (0%)	0/0 (0%)	2/4 (50%)	2/4 (50%)
	<300	3/6 (50%)	4/6 (67%)	4/7 (57%)	4/7 (57%)

NB-mRNA, neuroblastoma-associated mRNA; HR, high risk; PB, peripheral blood; BM, bone marrow; § 7NB-mRNAs ≥ 5.7, # 7NB-mRNAs ≥ 4.3, † 7NB-mRNAs ≥ 65.0, ‡ 7NB-mRNAs ≥ 4194.7; VMA, vanillylmandelic acid; HVA, homovanillic acid; NSE, neuron-specific enolase; LDH, lactate dehydrogenase.

## Data Availability

The data generated and analyzed in this study are available upon reasonable request.
